# Nursing competencies in aeromedical transport in the Brazilian Air Force: a descriptive study

**DOI:** 10.1590/1980-220X-REEUSP-2024-0129en

**Published:** 2024-08-12

**Authors:** Leticia Lima Borges, Beatriz Gerbassi Costa Aguiar, Débora Fernanda Haberland, Clarissa Coelho Vieira Guimarães, Rafael Abrantes de Lima, Bianca Beatriz Silva de Souza

**Affiliations:** 1 Universidade Federal do Estado do Rio de Janeiro, Rio de Janeiro, RJ, Brazil.; 2 Universidade Federal do Rio de Janeiro, Rio de Janeiro, RJ, Brazil.

**Keywords:** Nursing, Nurse’s Role, Aerospace Medicine, Enfermería, Rol de la Enfermera, Medicina Aeroespacial

## Abstract

**Objective:**

To analyze nursing skills in military aeromedical transport of the Brazilian Air Force.

**Method:**

Descriptive, qualitative research, carried out in three Brazilian Air Force hospitals in Rio de Janeiro, involving 64 military nurses. Discursive textual analysis identified competencies in healthcare, communication and decision-making.

**Results:**

After characterizing participants, it was possible to understand the reality experienced by nursing professionals in air medical transport, highlighting the skills related to healthcare, communication and management, essential in all phases of air medical transport.

**Conclusion:**

It was evident that nursing assumes unique skills in caring for airborne patients, using diverse knowledge and experiences in solving problems encountered in the aeromedical work process. The need to implement continuing education strategies was also highlighted. The findings serve as support for professionals and managers to identify gaps in knowledge, performance and management of professional schedules in aeromedical transport.

## RESUMEN

**Objetivo:**

Analizar las habilidades de enfermería en el transporte aeromédico militar de la Fuerza Aérea Brasileña.

**Método:**

Investigación descriptiva, cualitativa, realizada en tres hospitales de la Fuerza Aérea Brasileña en Río de Janeiro, con la participación de 64 enfermeros militares. El análisis textual discursivo identificó competencias en atención a la salud, comunicación y toma de decisiones.

**Resultados:**

Después de caracterizar a los participantes, fue posible comprender la realidad vivida por los profesionales de enfermería en el transporte médico aéreo, destacando las habilidades relacionadas con el cuidado de la salud, la comunicación y la gestión, esenciales en todas las fases del transporte médico aéreo.

**Conclusión:**

Se evidenció que la enfermería asume habilidades únicas en el cuidado de los pacientes en el aire, utilizando diversos conocimientos y experiencias en la solución de los problemas encontrados en el proceso de trabajo aeromédico. También se destacó la necesidad de implementar estrategias de educación continua. Los hallazgos sirven de apoyo a profesionales y gestores para identificar lagunas en el conocimiento, desempeño y gestión de horarios profesionales en el transporte aeromédico.

## INTRODUCTION

The Brazilian National Curricular Guidelines (*Diretrizes Curriculares Nacionais*) for the nursing course^(1)^ establish the need for nurses to have the ability to know and intervene in health problems prevalent in the country, highlighting general and specific competencies for different areas of activity. Competency involves the ability to “know how to act”, mobilize resources, integrate complex knowledge and have a strategic vision^(2)^.

Within this context, it is essential that nursing professionals develop competencies that guarantee comprehensive care, quality and humanization of care, including in air medical transport.

In Brazil, air medical transport had its first record in 1950, in the North region, in Belém, with the creation of Search And Rescue (SAR), through which the Brazilian Air Force (FAB – *Força Aérea Brasileira*) carried out search and rescue operations related to plane crashes^(3)^.

Since then, FAB has had important relevance in the development of humanitarian actions with populations in social vulnerability, whether in air transport of patients who are in need of care in remote locations or in rescuing the population in major environmental disasters.

In FAB’s organizational structure, the Air Force Health System (SISAU - *Sistema de Saúde da Aeronáutica*) has the mission of providing healthcare to Air Force Command military personnel, both active and reserve, pensioners and their dependents^(4)^.

Medical and hospital care within the scope of SISAU is interdependent and hierarchical, following criteria of increasing technical complexity, organized into four levels (1^st^, 2^nd^, 3^rd^ and 4^th^ levels) and regionalized in the five regions of Brazil.

In the state of Rio de Janeiro, there are three FAB hospitals: two classified as 4^th^ level hospitals (*Hospital de Força Aérea do Galeão* (HFAG) and *Hospital Central da Aeronáutica* (HCA)); and one classified as a 3^rd^ level hospital (*Hospital de Aeronáutica dos Afonsos* (HAAF)). Hospitalization and transfer of patients represent complementary activities in the system, and compliance with referral levels and standards is essential for the efficiency of SISAU and the best solution for patients^(5)^.

Due to the concentration of specialties and highly complex procedures in Rio de Janeiro, the State frequently receives patients transferred from other regions of the country. Due to Brazil’s vast territory, many of these transfers need to occur by air. In this way, the three hospitals, to fulfill the national aeromedical evacuation (AE) demands designated for the Rio de Janeiro area, work together, carrying out a monthly rotation, in which, each month, one of the three hospitals is responsible for carrying out AE and, therefore, will be responsible for scheduling military personnel who are on standby in aeromedical transport.

On the national scene, the Federal Nursing Council (Cofen – *Conselho Federal de Enfermagem*), understanding the practice of flight nursing, through Cofen Resolution 551 of 2017^(6)^, standardized the role of nurses in Mobile Pre-hospital and Inter-hospital care in fixed and rotary wing aircraft. With this resolution, it was established that, within the scope of nursing, it is exclusive to nurses to work in aeromedical transport. In 2020, Cofen updated this publication through Resolution 656, establishing aerospace nursing as an area of specialization that requires specific knowledge and skills for quality care.

The growth of Brazilian aeromedical activity in both quantity and complexity has become crucial to provide access to reference hospitals in large urban centers, especially for critical patients. In the context of FAB, nursing plays a fundamental role in all aeromedical transports, as established by the Aeronautics Command System Standard^(5)^.

Due to the complexity of this type of assistance, aerospace nursing emerges as an area of specialization that requires specific knowledge and skills. Competency consists of the mobilization of knowledge, skills and attitudes essential for carrying out a task. Therefore, we believe that identifying the competencies necessary for this type of transport will significantly contribute to nursing performance and the effective execution of related activities. In addition to the scientific aspects, it is essential that flight nurses are able to manage the entire transport, anticipating complications and providing the necessary resources during the flight.

In this context, this study aims to analyze nursing competencies in FAB military air medical transport. This analysis is fundamental, considering the importance of air medical transport in the Brazilian context, the growing demand for nursing professionals in this specific scenario and studies in this area are still incipient.

## METHOD

### Study Design

This is a descriptive study with a qualitative approach.

### Place

The study was carried out in three FAB hospitals, in the city of Rio de Janeiro, which carry out inter-hospital air medical transport within the scope of SISAU.

### Sample

Study participants were military nursing professionals who work in inter-hospital AE of three military hospitals in the city of Rio de Janeiro, such as HFAG, HCA and HAAF.

Military nursing officers and nursing technicians (1^st^, 2^nd^, 3^rd^ sergeants and non-commissioned officers) of the nursing service (SEF), who are designated to make up the AE scale of these hospitals were included. Military personnel who were on regulatory vacation, medical leave during the period defined for data collection and civilians in professional nursing practice were excluded.

In the three research scenarios, the universe of nursing officers and nursing technicians who participated in the AE scales of their respective Military Organizations was countless times greater than the number of participants interviewed. However, to maintain scientific and methodological rigor during data collection, it was decided to apply the theoretical saturation technique. Therefore, the “n” of the research was defined by observing the repetition of statements referring to the activities carried out by participants during air medical transport.

### Data Collection

Data collection was carried out through semi-structured interviews during September and October 2021. Before the interviews, prior contact was made with the immediate supervisor, with the aim of informing them of the study and verifying the best possibility of access to participants. Individually, each of them was informed about the study objective and purpose. After clarifying their doubts, they received the Informed Consent Form (ICF), and those who agreed to participate signed and answered the interview. Participation was voluntary, free of charge, and there was no reward for joining the study.

From the data collected during the interviews, it was possible to build participants’ profile with data such as age, sex, education, list belonging to FAB, length of military service, among others, these elements being important for understanding the social context, communication and access to information. These characteristics were essential for the researcher to know information that demonstrates the particularities of the sample to be studied, thus being able to understand the results in data analysis.

To construct the study on the competencies developed by the nursing team that works in FAB air medical transport, three questions were asked separately, one of which was: what activities are carried out by the nursing team pre-boarding during the flight and post-flight? The analysis of the answers highlighted several activities developed that imply the development of nursing competencies.

All interviews were audio recorded, with participants’ prior authorization, using a recorder device (MP3 type) and subsequently transcribed for analysis. In the transcriptions, participants were identified with codes (Participant = P), followed by Arabic numbers, guaranteeing their anonymity.

### Data Analysis and Treatment

The processing of the collected data followed Moraes and Galiazzi’s discursive textual analysis methodological framework, which constitutes a research methodology used to describe and interpret the content of all types of documents and texts. This analysis, conducting systematic descriptions, qualitative or quantitative, helps to reinterpret messages and achieve an understanding of their meanings at a level that goes beyond an ordinary reading^(7)^.

Initially, with the information collected, participants were characterized and, subsequently, the texts were deconstructed into a “*corpus*”, unitarization; the establishment of relationships between unitary elements, categorization; and emerging gathering, in which new understanding is communicated and validated.

With unitarization, it was possible to define the most frequent expressions that represent some feeling in relation to the duties carried out by nursing at each stage of the flight and, therefore, brought relevant information. By gathering, combining and classifying fragmented words according to a meaning relevant to the study purpose, it was possible to observe the emergence of categories of nursing competencies related to healthcare, communication and decision-making in air medical transport.

From the methodology presented, the three categories of the study emerged: Nursing competencies related to healthcare; Nursing competencies related to communication; and Nursing competencies related to management.

### Ethical Aspects

The study was submitted for consideration by the *Universidade Federal do Estado do Rio de Janeiro* Research Ethics Committee, being approved under Opinion 4,933,227 of August 26, 2021.

In order to meet ethical aspects, the present study followed what is recommended by Resolutions 466/2012 and 510/2016 of the Brazilian National Health Council.

All study participants had their rights guaranteed, by clarifying the objectives and proposed method and signing the ICF.

The research in question followed the *Universidade Federal do Estado do Rio de Janeiro* and FAB research standards, upon request for authorization from Military Organizations that carry out aeromedical transport.

## RESULTS

### Participant Characterization

The characterization of the 64 study participants allowed us to understand and highlight the reality experienced by nursing professionals who work in aeromedical transport, as shown in Table 1.

**Table 1:** Distribution of participants according to study variables carried out in three military hospitals of the Brazilian Air Force – Rio de Janeiro, RJ, Brazil, 2021.

Variables	n	(%)
Military Organization		
Hospital de Força Aérea do Galeão	19	29.7
Hospital Central da Aeronáutica	21	32.8
Hospital de Aeronáutica dos Afonsos	24	37.5
Age group		
20 to 30 years	17	26.5
31 to 40 years	36	56.2
41 to 45 years	08	12.5
Above 45 years	03	4.6
Sex		
Male	10	15.6
Female	54	84.4
List of the Brazilian Air Force that belongs		
List of Noncommissioned Officers and Sergeants	51	79.7
List of 2^nd^ Class Reserve Sergeants Called Up	10	15.6
List of 2^nd^ Class Reserve Officers Called Up	03	4.7
Force Support Officers	0	0
Education		
High school	27	42.2
Higher education	17	26.6
Specialization*	17	26.6
Master’s degree	02	3.1
Doctoral degree	01	1.5
Work shift		
12-hour day shift	27	42.2
12-hour night shift	19	29.7
Day laborers	18	28.1
Length of military service		
Up to 2 years	05	7.7
From 3 to 5 years	12	18.7
From 6 to 10 years	12	18.7
From 11 to 15 years	26	40.5
From 16 to 20 years	05	7.7
Over 20 years	05	7.7
Carrying out aeromedical evacuations in the military career		
Yes	38	59.4
No	26	20.6
Has another employment relationship		
Yes	13	20.3
No	51	79.7
Did some training to carry out air medical transport		
Yes	32	48.4
No	33	51.6

Note: *This is continuing education according to participants’ degree.

Regarding interviewees’ age, there was a predominance of participants between 31 and 40 years old. Among them, 54 participants were female and ten were male.

It was observed that 79.7% of respondents belong to the List of Noncommissioned Officers and Sergeants (career nursing technicians), 15.6% belong to the List of 2^nd^ Class Reserve Sergeants Called Up (temporary nursing technicians), and 4.7%, to the List of 2^nd^ Class Reserve Officers Called Up (temporary nurses). No participant belonged to the Force Support Officers (career nurse).

Concerning participants’ educational level, the research revealed that 42.2% completed high school; 26.6% studied until graduation; and the same 26.6% had graduate degrees. In the survey, only 3.1% had a master’s degree, and 1.5% completed a doctoral degree.

It was also asked whether research participants had already carried out any air medical transport in their careers, and it was shown that 59.4% had already participated, and 40.6%, despite competing for the scale, had not yet carried out air medical transport.

In relation to prior training for carrying out aeromedical transport, 48.4% of participants reported having had training and preparation for such an activity, with the Aeromedical Evacuation Course (AEC) being the main one.

The analysis of the results of this study allowed us to identify three nursing competencies developed in aeromedical transport, described below.

### Nursing Competencies Related to Healthcare

This competency highlighted both the care provided directly to patients during aeromedical transport, ensuring the integrity, safety and monitoring of clinical parameters as well as the assertiveness and organization necessary to prepare the aircraft according to needs. The following stands out in participants’ speeches:

*Everything is patient-focused, from start to finish. We have to take patients to their destination safely, ensuring that they have all the necessary support until they are delivered to the destination hospital* [...] (P41).

*During pre-boarding, our responsibility begins with separating the material, choosing the equipment that will be taken and preparing the aircraft, always aiming for individualized assistance.* [...] (P04).

Numerous participants also highlighted the importance of stabilizing patients before the flight so that, during transport, the team is only concerned with keeping them stable, as seen in the answers below:

*During the flight, it would basically be the maintenance of patients’ clinical condition and some intervention, if patients decompensate. But in pre-boarding, we already arrange everything so that this is not necessary. The less intervention during transport, the better for the team* […] (P02).

*Observe the hemodynamic stability of patients after transfer to the aircraft equipment and do everything necessary, while still on the ground, to maintain this stability during transport* [...] (P18).

### Nursing Competencies Related to Communication

This competency demonstrates the importance given by the aeromedical crew to maintaining effective and safe communication. Aviation imposes some communication difficulties that must be faced by the entire team. It is an environment with a high noise level, with a confined space, which brings together, on the same mission, health and aviation professionals who, therefore, have different languages, knowledge and objectives during the flight.

The aeromedical team’s concern with communication occurs both with the flight crew and with patients, family members on board and teams at the origin and destination units. Therefore, nursing faces major challenges in maintaining communication on board, which can be identified in participants’ statements below:

[...] *It is important to pay attention to the doctor during the mission, as it may be necessary to communicate even through gestures, as the noise is very loud on the aircraft* [...] (P41).

*Knowing the aircraft is important to be able to be agile in emergencies, in the procedures for a forced landing, all these issues are addressed in the briefing before the flight* [...] (P32).

### Nursing Competencies Related to Management

In the development of this competency, the nursing team is observed to be committed to the macro and micro aspects of air medical transport management. Macrospace management is dedicated to configuring the aircraft to meet the care needs of each patient as well as controlling and maintaining equipment, forecasting and supplying inputs. In this way, the development of management competencies in the macro space of aeromedical transport allows efficient planning so that the mission occurs without complications.

Microspace management deals with coordinated care provided to patients. It ranges from controlling materials and equipment used in checklist missions to the ability to manage hospital equipment on board and familiarization with the location of all supplies in the air medical transport backpack, as evidenced below in participants’ statements:

[...] *these are the precautions we must take for the flight, be sure to check the backpack checklist to know if we are taking all the necessary materials, all the medications* [...] (P52).

[...] *at the end of the mission, the team has to bring everything back, check it, see what was spent on replacing it, collect the equipment, such as the mechanical respirator, oximeter, infusion pump..., it is the responsibility of nursing in this return* [...] (P15).

## DISCUSSION

The results obtained indicate the predominance of female professionals, which reinforces the concept of nursing being carried out mainly by women, accounting for 89% of the profession, with variations between different regions of the world^(8)^. It is also possible to observe that air medical transport at FAB is carried out mainly by nursing technicians, corresponding to almost 80% of those interviewed.

It is noteworthy that approximately 50% of participants in this study report not having had prior training to compete for the AE scale. This result can be considered alarming, taking into account the work’s specificities and the recurring serious condition of patients chosen for air medical transport.

Among the participants who reported having had training for this activity, AEC was highlighted as the main one. To meet this need, this course is made available annually by the General Personnel Command (COMGEP - *Comando Geral de Pessoal*) in the Air Force Command Table (TCA (*Tabela do Comando da Aeronáutica*) 37-14/2022). This is a course that aims to qualify doctors, nurses and nursing technicians to work in AE in an operational and tactical environment. Taught by the Institute of Aerospace Medicine, it is structured on a theoretical-practical basis, in order to provide the knowledge and skills necessary during an AE^(9)^.

Assuming that excellence in training can increase flight safety, patient and team safety, in addition to promoting resource savings, reduced response time and quality in AE, all of these advantages can be achieved through training of health teams^(10)^.

The concept of competency is thought of as a set of knowledge, skills and attitudes that justify high performance, in which the best performances are based on people’s intelligence and personality^(11)^.

Among the competencies highlighted by the study, healthcare in aeromedical transport reveals individualized care that follows targeted and coordinated measures to maintain, at a minimum, the assistance provided in the unit of origin, aiming at promoting, protecting and maintaining stability of patients.

Separating all the necessary equipment for action and finding out about patients’ previous clinical status are important duties of nursing professionals, minimizing the possibility of unforeseen events during transport^(12)^. Therefore, it is observed that the pre-flight is a fundamental stage for successful air medical transport, and nursing stands out.

Therefore, pre-flight patient screening is an essential initial step to the success of aeromedical transport. When learning about patients’ health status, the equipment in use, the medications administered, whether they are using vasoactive amines, whether they have undergone any surgery, whether any transfusion was necessary, vital data and evolutionary hemodynamic status, the team will take this information as a basis for safe transport preparation by the aeromedical team^(13)^. Therefore, the ability to operate all of these precedents accurately is crucial for stabilization, treatment and patient safety during air transport.

During the flight, participants highlighted the care provided directly to patients, such as: keeping them hemodynamically stable; promoting their comfort (since the physiological conditions inherent to aviation can generate discomfort for patients, such as a drop in temperature, flight hypoxia, high noise levels and dysbarism); and keeping them safe (checking all circuits and drains and properly securing patients to the stretcher, preventing falls and securing all equipment securely to their bases).

When nursing procedures take place inside an aircraft, they encounter several adverse situations in relation to procedures carried out in a hospital environment, such as altitudes that vary from 500 to 5,000 feet above the ground, generating a hypobaric environment, unstable weather conditions and constant noise^(14)^.

It should also be noted that, in general, aircraft used for aeromedical transport have a confined space and a reduced team, which limits in-flight care. Therefore, after stabilization, during air medical transport, the team closely observes patients’ hemodynamic parameters, paying attention to any changes that occur. Therefore, the nursing team needs to know the aircraft used in air transport and understand the use of related medical equipment^(15,16,17)^.

The study also highlighted the concern that the nursing team has with the measures that provide comfort in flight, pain control and emotional support to patients and their families, when applicable. Speeches such as keeping patients properly warm, with adequate coverings and controlling the ambient temperature were raised, always aiming to avoid hypothermia, a common condition in flight.

The results of this study also revealed nursing competencies related to communication. Communication is defined as a social practice that arises from the interaction between human beings, expressed through verbal aspects, writing, gestural behaviors, the distance between participants and non-verbal aspects^(18)^. Therefore, communication in healthcare environments is complex and dynamic, and must be effective both between team professionals and patients, being essential for the adequate development of nursing work^(19)^.

In the process of efficient communication during the flight, it is essential that the aeromedical team communicates, interacts and becomes closer to the flight crew. In this process, each member will have a specific role on board, however aspects of flight safety, patient safety and biosafety must be shared by everyone involved in the transport. The working relationships between members of aeromedical transport teams highlight the ability to integrate multidisciplinary actions and establish effective communication^(20,21,22)^.

During a flight, the high volume of noise on aircraft can create difficulties in carrying out tasks that require interaction and coordination between people, making communication challenging. Such communication barriers require viable solutions to solve common problems, such as pain management in airborne patients^(23)^.

Another moment that demands close communication between the aeromedical team and the flight team is in the occurrence of unexpected events, such as in-flight emergencies. Communication between both teams must be based on previously trained and briefed decision-making. Therefore, holding a quick meeting before the start of the mission (briefing) was highlighted by several participants as facilitating communication and sharing information relevant to the flight^(24)^.

The study also revealed the nursing team’s management competency in aeromedical transport, evident both in the management of care provided directly to patients and in the development of activities related to planning, organization and provision of resources for all stages of the flight. Therefore, to exercise them, professionals need reflection, theoretical foundation and systematization that will help them analyze the conduct and decisions to be taken.

The decision to remove a patient is the responsibility of medical professionals, however it is up to nursing to meet all the necessary conditions in order to minimize the risks inherent to transport^(3)^. Therefore, nurses, as members of the multidisciplinary team that transports patients by air, are faced with challenges that require competencies that support them in decision-making in different situations^(25)^.

It was possible to observe that the nursing team is responsible for forecasting and providing materials, supplies and equipment prepared for the management of aeromedical service assistance, including checking rescue bags, checking expiration dates on supplies and medicines as well as checking the functionality of on-board equipment. Therefore, care management carried out by the onboard nursing team is fundamental to the care and administrative processes of aeromedical transport.

The team’s concern was also highlighted in recording the assistance provided in flight, committing to filling out forms and reports, essential for reducing failures in the process and reducing possible losses in understanding patients’ vital information, compromising continuity of care of nursing. The team must have good oral and written communication to discuss cases with the team and carry out the necessary reports^(10,26)^.

Although the study provided valuable insights into nursing competencies in FAB air medical transport, some limitations must be considered. The research was carried out only in three FAB hospitals, in Rio de Janeiro, which may not represent the totality of the experiences of nursing professionals in different regions and contexts. Being a descriptive and qualitative research, the results are based on individual perceptions and experiences of participants, introducing subjectivity and limiting the generalization of findings.

## CONCLUSION

The study showed that nursing professionals who work in FAB air medical transport developed essential competencies, skills and attitudes in healthcare, communication and management at all stages of this type of transport, making the provision of this care safer.

For professionals to fully develop their competencies, knowledge acquired through information, study and individual preparation is essential, prerequisites that are highly demanded in aeromedical transport, a specialized and complex professional activity. The development of skills in aeromedical transport involves continuous training and practice, highlighting the importance of periodic investments in valuing human capital.

To mitigate risks, strict regulations, professional training and safety protocols are implemented in FAB air medical transport. Safety is a top priority, and risks are proactively managed to ensure safe flights and effective onboard service.

The study highlights nursing, assuming relevance and unique competencies in the care of airborne patients, using diverse knowledge and experiences to solve problems. It also points out the clear use of professional competencies related to healthcare, communication and management.

Study contributions include a detailed characterization of how nurses develop and apply their competencies in the context of military air medical transport, helping nursing professionals identify gaps in knowledge and practice, allowing for more effective and managed performance. Furthermore, it promotes the autonomy of nurses, strengthening their ability to provide a high quality service and paving the way for future research on nursing competencies in air medical transport both in the military and in civil services.

The findings of this study can serve as support for professionals and managers in identifying and filling gaps in the knowledge, performance and management of professionals scheduled to perform AE, strengthening professional autonomy and the quality of the service provided. Furthermore, the results may open scope for new research on nursing competencies developed in aeromedical transport, both internally at FAB and externally in civil aeromedical transport services in the private or public network. Greater knowledge and integration of research on the subject aim to improve aeromedical activity and, consequently, improve the healthcare service provided to patients.
