# Supplementary material for: Nursing competencies in aeromedical transport in the Brazilian Air Force: a descriptive study
Source: Rev Esc Enferm USP. 2024 Aug 12;58:e20240129. doi: 10.1590/1980-220X-REEUSP-2024-0129en (PMC11321455; doi:10.1590/1980-220X-REEUSP-2024-0129en)
Supplement: Translation

# Translation: As competências de enfermagem no transporte aeromédico na Força Aérea Brasileira: estudo descritivo

**DOI:** 10.1590/1980-220X-REEUSP-2024-0129pt

**Authors:** Leticia Lima Borges, Beatriz Gerbassi Costa Aguiar, Débora Fernanda Haberland, Clarissa Coelho Vieira Guimarães, Rafael Abrantes de Lima, Bianca Beatriz Silva de Souza

**Affiliations:** 1 Universidade Federal do Estado do Rio de Janeiro, Rio de Janeiro, RJ, Brasil.; 2 Universidade Federal do Rio de Janeiro, Rio de Janeiro, RJ, Brasil.

**Keywords:** Enfermagem, Papel do Profissional de Enfermagem, Medicina Aeroespacial

## Abstract

**Objetivo:**

Analisar as competências de enfermagem no transporte aeromédico militar da Força Aérea Brasileira.

**Método:**

Pesquisa descritiva, qualitativa, realizada em três hospitais da Força Aérea Brasileira no Rio de Janeiro, envolvendo 64 militares de enfermagem. A análise textual discursiva identificou competências em atenção à saúde, comunicação e tomada de decisões.

**Resultados:**

Após a caracterização dos participantes, foi possível compreender a realidade vivenciada pelo profissional de enfermagem no transporte aeromédico, destacando as competências relacionadas à atenção à saúde, comunicação e gerenciamento, essenciais em todas as fases do transporte aeromédico.

**Conclusão:**

Evidenciou-se que a enfermagem assume competências singulares no cuidado aos pacientes aerotransportados, utilizando diversos conhecimentos e experiências na resolução de problemas encontrados no processo de trabalho aeromédico. Ressaltou-se também a necessidade de implementar estratégias de educação permanente. Os achados servem de subsídio para profissionais e gestores identificarem lacunas no conhecimento, na atuação e no gerenciamento das escalas de profissionais no transporte aeromédico.

## INTRODUÇÃO

As Diretrizes Curriculares Nacionais do curso de enfermagem^(1)^ estabelecem a necessidade de que os enfermeiros tenham a capacidade de conhecer e intervir em problemas de saúde prevalentes no país, destacando competências gerais e específicas para diversas áreas de atuação. A competência envolve a habilidade de “saber agir”, mobilizar recursos, integrar saberes complexos e ter visão estratégica^(2)^.

Dentro desse contexto, é essencial que os profissionais de enfermagem desenvolvam competências que garantam a integralidade da atenção, qualidade e humanização do atendimento, inclusive no transporte aeromédico.

No Brasil, o transporte aeromédico teve seu primeiro registro em 1950, na Região Norte, em Belém, a partir da criação do Serviço de Busca e Salvamento (SAR, do inglês, *Search And Rescue*), por meio do qual a Força Aérea Brasileira (FAB) realizava a busca e salvamentos relacionados a acidentes aéreos^(3)^.

Desde então, a FAB tem importante relevância no desenvolvimento de ações humanitárias junto às populações em vulnerabilidade social, seja no transporte aéreo de pacientes que estejam necessitando de atendimento em locais remotos ou nas atuações de resgates da população em grandes desastres ambientais.

Na estrutura organizacional da FAB, o Sistema de Saúde da Aeronáutica (SISAU) tem a missão de prestar assistência de saúde aos militares do Comando da Aeronáutica, tanto na ativa quanto na reserva, pensionistas e seus dependentes^(4)^.

Os atendimentos médico-hospitalares no âmbito do SISAU são interdependentes e hierarquizados, seguindo critérios de complexidade técnica crescente, organizados em quatro escalões (1º, 2º, 3º e 4º escalões) e regionalizados nas cinco regiões do Brasil.

No estado do Rio de Janeiro, há três hospitais da FAB: dois classificados como hospitais de 4º escalão (Hospital de Força Aérea do Galeão (HFAG) e Hospital Central da Aeronáutica (HCA)); e um classificado como hospital de 3º escalão (Hospital de Aeronáutica dos Afonsos (HAAF)). A hospitalização e a transferência de pacientes representam atividades complementares no sistema, e a observância dos escalões e normas de encaminhamento é essencial para a eficiência do SISAU e a melhor solução para os pacientes^(5)^.

Devido à concentração de especialidades e procedimentos de alta complexidade no Rio de Janeiro, o Estado frequentemente recebe pacientes transferidos de outras regiões do país. Devido à vastidão territorial do Brasil, muitas dessas transferências precisam ocorrer por meio aéreo. Dessa forma, os três hospitais, para cumprirem as demandas de evacuações aeromédicas (EVAM) nacionais designadas para a área do Rio de Janeiro, trabalham juntos, realizando um rodízio mensal, no qual, a cada mês, um dos três hospitais é o responsável por realizar as EVAM e, portanto, será o responsável por escalar os militares que estão de sobreaviso nos transportes aeromédicos.

No cenário nacional, o Conselho Federal de Enfermagem (Cofen), entendendo a prática da enfermagem de voo, por meio da Resolução Cofen nº 551 de 2017^(6)^, normatizou a atuação do enfermeiro no atendimento Pré-hospitalar Móvel e Inter-hospitalar em Aeronaves de asa fixa e rotativa. Com essa resolução, ficou estabelecido que, no âmbito da enfermagem, é privativa ao enfermeiro a atuação em transportes aeromédicos. Em 2020, o Cofen atualizou essa publicação por meio da Resolução nº 656, estabelecendo a enfermagem aeroespacial como uma área de especialização que requer conhecimentos e habilidades específicas para uma assistência de qualidade.

O crescimento da atividade aeromédica brasileira tanto em quantidade quanto em complexidade se tornou crucial para proporcionar acesso a hospitais de referência em grandes centros urbanos, especialmente para pacientes críticos. No contexto da FAB, a enfermagem desempenha um papel fundamental em todos os transportes aeromédicos, conforme estabelecido pela Norma do Sistema do Comando da Aeronáutica^(5)^.

Devido à complexidade desse tipo de assistência, a enfermagem aeroespacial surge como uma área de especialização que exige conhecimentos e habilidades específicas. A competência consiste na mobilização de conhecimentos, habilidades e atitudes essenciais para a realização de uma função. Dessa forma, acreditamos que identificar as competências necessárias para esse tipo de transporte contribuirá significativamente para a atuação da enfermagem e para a execução eficaz das atividades relacionadas. Além dos aspectos científicos, é fundamental que o enfermeiro de voo seja capaz de gerenciar todo o transporte, antecipando intercorrências e fornecendo os recursos necessários durante o voo.

Nesse contexto, este estudo tem como objetivo analisar as competências de enfermagem no transporte aeromédico militar da FAB. Essa análise é fundamental, considerando a importância do transporte aeromédico brasileiro, a crescente demanda por profissionais de enfermagem nesse cenário específico e os estudos incipientes nessa área.

## MÉTODO

### Tipo de Estudo

Trata-se de estudo descritivo com abordagem qualitativa.

### Local

O estudo desenvolveu-se em três hospitais da FAB, no município do Rio de Janeiro, que realizam transporte aeromédico inter-hospitalar no âmbito do SISAU.

### Amostra

Os participantes deste estudo foram militares profissionais de enfermagem que atuam na EVAM inter-hospitalar de três hospitais militares do município do Rio de Janeiro, tais como HFAG, HCA e HAAF.

O critério de inclusão utilizado neste estudo abrangeu militares oficiais enfermeiros e os técnicos de enfermagem (1º, 2º, 3º sargentos e suboficiais) do serviço de enfermagem (SEF), que são designados para compor a escala de EVAM desses hospitais. Utilizou-se como critério de exclusão os militares que estavam de férias regulamentares, licença médica no período definido para a coleta de dados e os civis em exercício profissional de enfermagem.

Nos três cenários da pesquisa, o universo de oficiais enfermeiros e técnicos de enfermagem que participavam das escalas de EVAM de suas respectivas Organizações Militares foi inúmeras vezes maior que o número de participantes entrevistados. No entanto, para manter o rigor científico e metodológico durante a coleta de dados, optou-se por aplicar a técnica de saturação teórica. Portanto, o “n” da pesquisa foi definido ao observar a repetição das falas referentes às atividades realizadas pelos participantes no transporte aeromédico.

### Coleta de Dados

A coleta de dados foi realizada por meio de entrevista semiestruturada durante os meses de setembro e outubro de 2021. Antes das entrevistas, foi realizado contato prévio com a chefia imediata, com o objetivo de dar ciência do estudo e verificar a melhor possibilidade de acesso aos participantes. Individualmente, cada um deles foi informado sobre o objetivo e a finalidade do estudo. Após o esclarecimento das dúvidas, receberam o Termo de Consentimento Livre e Esclarecido (TCLE), e aqueles que aceitaram participar assinaram e responderam à entrevista. A participação foi voluntária, sem ônus, e não houve qualquer tipo de recompensa pela adesão ao estudo.

A partir dos dados coletados durante as entrevistas, foi possível a construção do perfil dos participantes com dados como idade, sexo, escolaridade, quadro que pertence na FAB, tempo de serviço militar, entre outros, sendo esses elementos importantes para a compreensão do contexto social, comunicação e acesso a informações. Essas características foram essenciais para o pesquisador conhecer informações que demonstrem as particularidades da amostra a ser estudada, compreendendo os resultados na análise dos dados.

Para a construção do estudo sobre as competências desenvolvidas pela equipe de enfermagem que atua nos transportes aeromédicos da FAB, foram realizadas três perguntas separadamente, sendo uma delas: quais as atividades são realizadas pela equipe de enfermagem no pré-embarque durante o voo e no pós-voo A análise das respostas evidenciou diversas atividades desenvolvidas que implicam o desenvolvimento de competências de enfermagem.

Todas as entrevistas foram gravadas em áudio, com a autorização prévia do participante, por meio de aparelho de gravador (tipo MP3) e, posteriormente, transcritas para análise. Nas transcrições, os participantes foram identificados com os códigos (Participante = P), seguidos de números arábicos, garantindo seu anonimato.

### Análise e Tratamento dos Dados

O processamento dos dados coletados seguiu o referencial metodológico da análise textual discursiva de Moraes e Galiazzi, que constitui uma metodologia de pesquisa usada para descrever e interpretar o conteúdo de toda classe de documentos e textos. Essa análise, conduzindo as descrições sistemáticas, qualitativas ou quantitativas, ajuda a reinterpretar as mensagens e atingir uma compreensão de seus significados em um nível que vai além de uma leitura comum^(7)^.

Em um primeiro momento, com as informações coletadas, ocorreu a caracterização dos participantes e, posteriormente, a desconstrução dos textos em “*corpus*”, a unitarização; o estabelecimento de relações entre os elementos unitários, a categorização; e o captar emergente, em que a nova compreensão é comunicada e validada.

Com a unitarização, foi definiu-se as expressões mais frequentes que representam algum sentimento em relação às atribuições desenvolvidas pela enfermagem em cada etapa do voo e, portanto, trouxeram informações relevantes. Ao reunir, combinar e classificar as palavras fragmentadas em função de um sentido pertinente ao propósito do estudo, foi possível observar a emergência de categorias das competências de enfermagem relacionadas à atenção à saúde, à comunicação e à tomada de decisões no transporte aeromédico.

A partir da metodologia apresentada, emergiram as três categorias do estudo: Competências de enfermagem relacionadas à atenção à saúde; Competências de enfermagem relacionadas à comunicação; e Competências de enfermagem relacionadas à gerência.

### Aspectos Éticos

O estudo foi submetido à apreciação do Comitê de Ética e Pesquisa da Universidade Federal do Estado do Rio de Janeiro, sendo aprovado sob Parecer nº 4.933.227, de 26 de agosto de 2021.

A fim de atender aos aspectos éticos, o presente estudo seguiu o que é preconizado pelas Resoluções nº 466/2012 e n° 510/2016 do Conselho Nacional de Saúde.

Todos os participantes do estudo tiveram seus direitos assegurados, mediante esclarecimento dos objetivos e do método proposto e a assinatura do TCLE.

A pesquisa em questão seguiu as normas de pesquisa da Universidade Federal do Estado do Rio de Janeiro e da FAB, mediante solicitação de autorização das Organizações Militares que realizam transporte aeromédico.

## RESULTADOS

### Caracterização dos Participantes

A caracterização dos 64 participantes do estudo permitiu compreender e evidenciar a realidade vivenciada pelo profissional de enfermagem que atua no transporte aeromédico, conforme demonstrada na Tabela 1.

**Tabela 1:** Distribuição dos participantes segundo as variáveis do estudo realizado em três hospitais militares da Força Aérea Brasileira – Rio de Janeiro, RJ, Brasil, 2021.

Variáveis	n	(%)
Organização Militar		
Hospital de Força Aérea do Galeão	19	29,7
Hospital Central da Aeronáutica	21	32,8
Hospital de Aeronáutica dos Afonsos	24	37,5
Faixa etária		
20 a 30 anos	17	26,5
31 a 40 anos	36	56,2
41 a 45 anos	08	12,5
Acima de 45 anos	03	4,6
Sexo		
Masculino	10	15,6
Feminino	54	84,4
Quadro da Força Aérea Brasileira que pertence		
Quadro de Suboficiais e Sargentos	51	79,7
Quadro de Sargentos da Reserva de 2ª Classe Convocados	10	15,6
Quadro de Oficiais da Reserva de 2ª Classe Convocados	03	4,7
Quadro de Oficiais de Apoio da Aeronáutica	0	0
Escolaridade		
Nível médio	27	42,2
Nível superior	17	26,6
Especialização*	17	26,6
Mestrado	02	3,1
Doutorado	01	1,5
Turno de trabalho		
Plantão 12 horas diurnas	27	42,2
Plantão 12 horas noturnas	19	29,7
Diaristas	18	28,1
Tempo de serviço militar		
Até 2 anos	05	7,7
De 3 a 5 anos	12	18,7
De 6 a 10 anos	12	18,7
De 11 a 15 anos	26	40,5
De 16 a 20 anos	05	7,7
Acima de 20 anos	05	7,7
Realização de evacuações aeromédicas na carreira militar		
Sim	38	59,4
Não	26	20,6
Possui outro vínculo empregatício		
Sim	13	20,3
Não	51	79.7
Fez alguma capacitação para realizar transporte aeromédico
Sim	32	48,4
Não	33	51,6

Nota: *Trata-se de educação continuada segundo a graduação do participante.

Em relação à idade dos entrevistados, constatou-se uma predominância de participantes na faixa etária entre 31 e 40 anos. Entre eles, 54 participantes eram do sexo feminino e dez eram do sexo masculino.

Observou-se que 79,7% dos entrevistados pertencem ao Quadro de Suboficiais e Sargentos (técnicos de enfermagem de carreira), 15,6%, ao Quadro de Sargentos da Reserva de 2ª Classe Convocados (técnicos de enfermagem temporários), e 4,7%, ao Quadro de Oficiais da Reserva de 2ª Classe Convocados (enfermeiros temporários). Nenhum participante pertencia ao Quadro de Oficiais de Apoio da Aeronáutica (enfermeiro de carreira).

Com relação ao nível de escolaridade dos participantes, a pesquisa revelou que 42,2% cursaram até o nível médio; 26,6% cursaram até a graduação; e os mesmos 26,6% tinham pós-graduação. Na pesquisa, apenas 3,1% tinham mestrado, e 1,5% cursaram até o doutorado.

Foi questionado ainda se os participantes da pesquisa já haviam realizado algum transporte aeromédico em suas carreiras, e foi evidenciado que 59,4% já haviam participado, e 40,6%, apesar de concorrem à escala, ainda não tinham realizado transporte aeromédico.

Quanto à realização de capacitação prévia para a realização de transporte aeromédico, 48,4% dos participantes referiram ter tido treinamento e preparo para tal atividade, sendo o Curso de Evacuação Aeromédica (CEVAM) evidenciado como o principal.

A análise dos resultados deste estudo permitiu identificar três competências de enfermagem desenvolvidas no transporte aeromédico descritas abaixo.

### Competências de Enfermagem Relacionadas à Atenção à Saúde

Nessa competência, evidenciaram-se tanto o cuidado prestado diretamente ao paciente no transporte aeromédico, assegurando a integridade, a segurança e a monitorização dos parâmetros clínicos, quanto a assertividade e a organização necessárias para o preparo da aeronave de acordo com as necessidades. Destaca-se nas falas dos participantes:

*Tudo é voltado para o paciente, do início ao fim. Temos que levar o paciente ao seu destino com toda a segurança, garantindo que tenha todo suporte necessário até que seja entregue ao hospital de destino* [...] (P41).

*No pré-embarque, nossa responsabilidade inicia-se desde a separação do material, da eleição dos equipamentos que serão levados e do preparo da aeronave, sempre visando à assistência individualizada* [...] (P04).

Inúmeros participantes também ressaltaram a importância de estabilizar o paciente previamente ao voo, para que, durante o transporte, a equipe se preocupe apenas em mantê-los estáveis, conforme observa-se nas respostas abaixo:

*Durante o voo, seria basicamente a manutenção do quadro clínico do paciente e alguma intervenção, caso o paciente descompense. Mas no pré-embarque, a gente já acerta tudo para que isso não seja preciso. Quanto menos intervenção durante o transporte, melhor para a equipe* […] (P02).

*Observar a estabilidade hemodinâmica do paciente após a transferência para os equipamentos da aeronave e fazer tudo o que for necessário, ainda em solo, para manter esta estabilidade durante o transporte* [...] (P18).

### Competências de Enfermagem Relacionadas à Comunicação

Essa competência demonstra a importância dedicada pela tripulação aeromédica para manter uma comunicação efetiva e segura. A aviação impõe algumas dificuldades de comunicação que deverão ser enfrentadas por toda a equipe. Trata-se de um ambiente com alto índice de ruído, com espaço confinado, que reúne, em uma mesma missão, profissionais de saúde e aviação que, portanto, possuem linguagens, saberes e objetivos diferentes durante o voo.

A preocupação da equipe aeromédica com a comunicação ocorre tanto com a tripulação de voo quanto com os pacientes, os familiares a bordo e as equipes das unidades de origem e destino. Dessa forma, a enfermagem encontra grandes desafios na manutenção da comunicação a bordo, que podem ser identificados nas falas dos participantes abaixo:

[...] é importante ficar atento ao médico durante a missão, pois pode ser necessário se comunicar até por gestos, pois o barulho é muito alto na aeronave [...] (P41).

*Conhecer a aeronave é importante para poder ter agilidade em emergências, nos procedimentos de um pouso forçado, todas essas questões são tratadas no briefing antes do voo* [...] (P32).

### Competências de Enfermagem Relacionadas à Gerência

No desenvolvimento dessa competência, observa-se a equipe de enfermagem empenhada nos aspectos macro e micro do gerenciamento do transporte aeromédico. A gerência do macroespaço dedica-se à configuração da aeronave para atender às necessidades assistenciais de cada paciente, assim como realizar o controle e a manutenção dos equipamentos, a previsão e a provisão dos insumos. Dessa forma, o desenvolvimento das competências de gerência no espaço macro do transporte aeromédico permite o planejamento eficiente para que a missão ocorra sem intercorrências.

Já a gerência do microespaço trata da assistência coordenada prestada ao paciente. Ela engloba desde a realização do controle de materiais e equipamentos utilizados nas missões com as listas de verificação (*checklist*) até a habilidade no manejo dos equipamentos hospitalares a bordo e a familiarização com a localização de todos os insumos da mochila do transporte aeromédico, conforme evidenciado abaixo nas falas dos participantes da pesquisa:

[...] *são os cuidados que devemos ter para o voo, não deixar de observar o checklist da mochila para saber se estamos levando todos os materiais necessários, todas as medicações* [...] (P52).

[...] *ao término da missão, a equipe tem que trazer tudo de volta, conferir, ver o que gastou para repor, recolher os equipamentos, como o respirador mecânico, oxímetro, bomba infusora..., é responsabilidade da enfermagem nesse retorno* [...] (P15).

## DISCUSSÃO

Os resultados obtidos indicam a predominância de profissionais do sexo feminino, o que reforça o conceito da enfermagem ser exercida majoritariamente por mulheres, representando 89% do quantitativo da profissão, com variações entre as diferentes regiões do mundo^(8)^. É possível observar ainda que o transporte aeromédico na FAB é realizado majoritariamente por técnicos de enfermagem, correspondendo a quase 80% dos entrevistados.

Destaca-se que aproximadamente 50% dos participantes deste estudo referem não ter tido capacitação prévia para concorrer à escala de EVAM. Tal resultado pode ser considerado alarmante, levando-se em consideração as especificidades do trabalho e o recorrente estado de gravidade dos pacientes eleitos para o transporte aeromédico.

Entre os participantes que referiram ter tido treinamento para tal atividade, o CEVAM foi evidenciado como o principal. Para atender a essa necessidade, esse curso é disponibilizado anualmente pelo Comando Geral de Pessoal (COMGEP) na Tabela do Comando da Aeronáutica (TCA 37-14/2022). Trata-se de um curso que tem como finalidade habilitar médicos, enfermeiros e técnicos de enfermagem para atuarem nas EVAM em ambiente operacional e tático. Ministrado pelo Instituto de Medicina Aeroespacial, estrutura-se em uma base teórico-prática, a fim de prover conhecimentos e habilidades necessários durante uma EVAM^(9)^.

Partindo do princípio de que a excelência no treinamento pode aumentar a segurança do voo, a segurança do paciente e da própria equipe, além de promover a economia dos recursos, a redução do tempo de resposta e a qualidade nas EVAM, todas essas vantagens podem ser alcançados por meio da capacitação das equipes de saúde^(10)^.

O conceito de competência é pensado como um conjunto de conhecimentos, habilidades e atitudes que justificam alto desempenho, nos quais os melhores desempenhos estão fundamentados na inteligência e na personalidade das pessoas^(11)^.

Entre as competências evidenciadas pelo estudo, a assistência à saúde no transporte aeromédico revela-se um cuidado individualizado que segue medidas direcionadas e coordenadas para manter, minimamente, a assistência prestada na unidade de origem, objetivando a promoção, a proteção e a manutenção da estabilidade do paciente.

Separar todo o equipamento necessário para atuação e inteirar-se sobre o estado clínico prévio do paciente são atribuições importantes do profissional de enfermagem, minimizando a possibilidade de imprevistos durante o transporte^(12)^. Destarte, observa-se que o pré-voo é uma etapa fundamental para que o transporte aeromédico seja bem-sucedido, e tem como protagonista a atuação da enfermagem.

Portanto, a realização da triagem do paciente no pré-voo são etapas iniciais imprescindíveis ao sucesso do transporte aeromédico. Ao inteirar-se do estado de saúde do paciente, dos equipamentos em uso, dos medicamentos administrados, se está em uso de aminas vasoativas, se foi submetido a alguma cirurgia, se foi necessária alguma transfusão, os dados vitais e o estado hemodinâmico evolutivo, a equipe tomará essas informações como base para o preparo seguro do transporte pela equipe aeromédica^(13)^. Dessa forma, a capacidade de operar todos esses precedentes com precisão é crucial para a estabilização, o tratamento e a segurança do paciente durante o transporte aéreo.

Já durante o voo, os participantes evidenciaram os cuidados ministrados diretamente ao paciente como: mantê-lo estável hemodinamicamente; promover seu conforto (visto que as condições fisiológicas inerentes à aviação podem gerar desconforto no paciente, como queda da temperatura, hipóxia de voo, alto nível de ruído e disbarismo); e mantê-lo seguro (checando todos os circuitos e drenos e fixando adequadamente o paciente na maca, evitando quedas e afivelando bem todos os equipamentos em suas bases).

Os procedimentos de enfermagem, quando acontecem dentro de uma aeronave, encontram diversas situações adversas em relação aos procedimentos realizados em um ambiente hospitalar, como altitudes que variam de 500 a 5.000 pés em relação ao solo, gerando um ambiente hipobárico, condições climáticas instáveis e ruídos constantes^(14)^.

Destaca-se ainda que, em geral, as aeronaves utilizadas para os transportes aeromédico possuem um espaço confinado e uma equipe reduzida, o que limita os cuidados em voo. Dessa forma, após a estabilização, durante o transporte aeromédico, a equipe procede à observação rigorosa dos parâmetros hemodinâmicos do paciente, atentando-se para quaisquer alterações que aconteçam. Dessa forma, a equipe de enfermagem precisa conhecer as aeronaves utilizadas em transporte aéreo e compreender o uso de equipamentos médicos relacionados^(15–17)^.

Também foi evidenciada pelo estudo a preocupação que a equipe de enfermagem tem com as medidas que proporcionam conforto em voo, controle da dor e apoio emocional aos pacientes e seus familiares, quando houver. Falas como manter o paciente devidamente aquecido, com coberturas adequadas e fazer o controle da temperatura ambiente foram levantadas, sempre visando evitar a hipotermia, uma condição comum em voo.

Os resultados deste estudo revelaram ainda competências de enfermagem relacionadas à comunicação. A comunicação é definida como prática social que advém da interação entre seres humanos, expressa por meio dos aspectos verbais, a escrita, os comportamentos gestuais, a distância entre os participantes e aspectos não verbais^(18)^. Dessa forma, a comunicação nos ambientes da saúde é complexa e dinâmica, e deve ser efetiva tanto entre profissionais da equipe quanto com o paciente, sendo primordial para o adequado desenvolvimento do trabalho da enfermagem^(19)^.

No processo de comunicação eficiente durante o voo, é imprescindível que a equipe aeromédica comunique-se, interaja e aproxime-se da tripulação de voo. Nesse processo, cada integrante terá função específica a bordo, no entanto aspectos de segurança de voo, segurança do paciente e biossegurança devem ser compartilhados por todos os envolvidos no transporte. As relações de trabalho entre os membros das equipes de transporte aeromédico destacam a capacidade de integrar ações multiprofissionais e de se estabelecer efetiva comunicação^(20–22)^.

Durante o voo, o elevado volume de ruído nas aeronaves pode gerar dificuldades em realizar tarefas que requeiram interação e coordenação entre pessoas, tornando a comunicação desafiadora. Tais barreiras de comunicação exigem soluções viáveis para a resolução de problemas comuns, como o manejo da dor no paciente aerotransportado^(23)^.

Outro momento que demanda estreita comunicação entre a equipe aeromédica e a equipe de voo é na ocorrência de eventos inesperados, como nas emergências em voo. A comunicação de ambas as equipes deve ser baseada em tomada de decisão previamente treinada e briefada. Portanto, a realização de uma rápida reunião antes do início da missão (*briefing*) foi apontada por diversos participantes como facilitadora da comunicação e compartilhamento de informações pertinentes ao voo^(24)^.

O estudo revelou ainda a competência de gerência pela equipe de enfermagem no transporte aeromédico, evidenciado tanto no gerenciamento do cuidado prestado diretamente ao paciente quanto no desenvolvendo de atividades relacionadas ao planejamento, à organização e à provisão de recursos para todas as etapas do voo. Portanto, para exercê-las, o profissional necessita de reflexão, de fundamentação teórica e de sistematização que o auxiliarão na análise das condutas e nas decisões a serem tomadas.

A decisão de remover um paciente é de competência do profissional médico, no entanto compete à enfermagem reunir todas as condições necessárias, de modo a minimizar os riscos inerentes ao transporte^(3)^. Dessa forma, o enfermeiro, como membro da equipe multiprofissional que transporta pacientes por meio aéreo, defronta-se com desafios que requerem competências que o respaldem na tomada de decisão nas diversas situações^(25)^.

Foi possível observar que a equipe de enfermagem se responsabiliza desde a previsão e provisão de materiais, insumos e equipamentos preparados para a gestão da assistência do serviço aeromédico até a checagem das bolsas de resgate, conferência de datas de validade de insumos e medicamentos, assim como verificação da funcionalidade dos equipamentos de bordo. Portanto, gerenciamento do cuidado realizado pela equipe de enfermagem a bordo é fundamental para os processos assistenciais e administrativos do transporte aeromédico.

Foi destacada ainda a preocupação da equipe em registrar a assistência prestada em voo, comprometendo-se com o preenchimento de formulários e relatórios, imprescindíveis para a redução de falhas no processo e reduzindo possíveis prejuízos na compreensão de informações vitais do paciente, comprometendo a continuidade dos cuidados de enfermagem. A mesma deve possuir boa comunicação oral e escrita para discutir casos com a equipe e realizar os relatórios necessários^(10,26)^.

Embora o estudo tenha fornecido *insights* valiosos sobre as competências de enfermagem no transporte aeromédico da FAB, algumas limitações devem ser consideradas. A pesquisa foi realizada apenas em três hospitais da FAB, no Rio de Janeiro, o que pode não representar a totalidade das experiências dos profissionais de enfermagem em diferentes regiões e contextos. Sendo uma pesquisa descritiva e qualitativa, os resultados são baseados em percepções e experiências individuais dos participantes, introduzindo subjetividade e limitando a generalização dos achados.

## CONCLUSÃO

O estudo mostrou que os profissionais de enfermagem que atuam no transporte aeromédico da FAB desenvolveram competências, habilidades e atitudes essenciais em assistência à saúde, comunicação e gerência em todas as etapas desse tipo de transporte, tornando mais segura a prestação desse cuidado.

Para que os profissionais desenvolvam plenamente suas competências, é imprescindível o conhecimento adquirido por meio da informação, do estudo e do preparo individual, pré-requisitos altamente exigidos no transporte aeromédico, uma atividade profissional especializada e complexa. O desenvolvimento das habilidades no transporte aeromédico envolve contínuo treinamento e prática, evidenciando a importância de investimentos periódicos na valorização do capital humano.

Para mitigar os riscos, regulamentos rigorosos, treinamento profissional e protocolos de segurança são implementados no transporte aeromédico da FAB. A segurança é prioridade máxima, e os riscos são gerenciados de forma proativa para garantir voos seguros e atendimento eficaz a bordo.

O estudo evidencia a enfermagem, assumindo relevância e competências singulares no cuidado aos pacientes aerotransportados, utilizando diversos conhecimentos e experiências para a resolução de problemas. Aponta ainda o nítido emprego das competências profissionais relacionadas à assistência à saúde, comunicação e gerência.

Contribuições do estudo incluem a caracterização detalhada sobre como os enfermeiros desenvolvem e aplicam suas competências no contexto do transporte aeromédico militar, auxiliando profissionais de enfermagem a identificar lacunas no conhecimento e na prática, permitindo uma atuação mais eficaz e gerenciada. Além disso, promove a autonomia dos enfermeiros, fortalecendo sua capacidade de prestar um serviço de alta qualidade e abrindo caminho para futuras pesquisas sobre as competências de enfermagem no transporte aeromédico tanto no âmbito militar quanto em serviços civis.

Os achados deste estudo podem servir de subsídio para profissionais e gestores na identificação e no preenchimento de lacunas no conhecimento, na atuação e na gestão dos profissionais escalados para realizar EVAM, fortalecendo a autonomia profissional e a qualidade do serviço prestado. Além disso, os resultados poderão abrir escopo para novas pesquisas sobre as competências de enfermagem desenvolvidas no transporte aeromédico, tanto no cenário interno da FAB quanto externamente nos serviços de transporte aeromédico civis da rede privada ou pública. O maior conhecimento e a integração de pesquisas sobre a temática têm o objetivo de aperfeiçoar a atividade aeromédica e, consequentemente, melhorar o serviço de saúde prestado aos pacientes.
